# Sex differences in the impact of extreme heat on cardiovascular disease outcomes: a systematic review and meta-analysis

**DOI:** 10.1186/s12940-025-01175-6

**Published:** 2025-04-12

**Authors:** Yusheng Zhou, Léa Larochelle, Fahima Afsari Khan, Louise Pilote

**Affiliations:** 1 Centre for Outcomes Research and Evaluation, Research Institute of McGill University Health Centre, 5252 De Maisonneuve Blvd, QC H4A 3S5 Montreal, Canada; 2 Faculty of Medicine and Health Sciences, McGill University, 3605 Rue de la Montagne, QC H3G 2M1 Montreal, Canada

## Abstract

**Background:**

Climate change is intensifying extreme heat events, posing significant risks to cardiovascular health. While sex differences in heat vulnerability have been observed, the evidence remains inconsistent. This systematic review and meta-analysis examined sex-specific associations between extreme heat exposure and cardiovascular disease (CVD) outcomes over the past decade.

**Methods:**

We searched PubMed, Embase, and Scopus for studies published between 2004 and 2024 that reported sex-stratified cardiovascular outcomes associated with heat exposure following the PRISMA guidelines. The quality of the evidence was evaluated following the Navigation Guide Criteria. Random-effects meta-analysis was conducted to calculate pooled relative risk ratios (RRR) comparing males to females for studies addressing incremental temperature increase. Heat wave studies were synthesized narratively due to methodological heterogeneity.

**Results:**

Of 6126 articles, 79 met inclusion criteria (62 in meta-analysis, 17 in narrative synthesis), primarily from East Asia, Europe, and North America. A 1 °C temperature increase was associated with elevated cardiovascular risks for both sexes. The pooled relative risk ratio (RRR) comparing males to females was 1.008 [1.002–1.014] for mortality, suggesting slightly higher female vulnerability, but not for morbidity (RRR 0.996 [0.987–1.004]). Significant heterogeneity was noted (Mortality I² = 50.3%, Morbidity I² = 70.3%). Heat wave studies showed inconsistent sex-specific impacts across populations.

**Conclusions:**

Females showed marginally higher vulnerability to heat-related cardiovascular mortality compared to males, while no significant sex differences were observed for morbidity outcomes. Future research should focus on understanding these mechanisms and developing sex-specific interventions.

**Supplementary Information:**

The online version contains supplementary material available at 10.1186/s12940-025-01175-6.

## Introduction

As climate change intensifies, extreme heat events are becoming more frequent, intense, and prolonged, posing a significant threat to global health [1, 2]. The burden of disease and mortality attributable to global warming is steadily rising [3–5], with approximately 489,000 heat-related deaths annually from 2000 to 2019 [6] and 153,000 heat wave-related deaths during each warm season from 1990 to 2019 [7]. Specifically, rising ambient temperature significantly exacerbates the risk of cardiovascular disease(CVD), contributing to a substantial number of deaths and place a significant burden on healthcare systems globally [5, 8, 9]. In 2019 alone, heat contributed to around 90,000 CVD-related deaths worldwide [8]. The strong correlation between extreme temperatures and cardiovascular health emphasizes the urgent need to identify factors that render certain population groups more vulnerable to heat, thereby enabling the implementation of adequate and effective preventive measures at both the individual and societal levels.

In epidemiological studies of heat-related cardiovascular outcomes, sex is increasingly recognized as a critical vulnerability factor, where vulnerability refers to a greater likelihood of adverse outcomes given a specific exposure, compared with the general population [9]. Sex modifies cardiovascular responses to heat exposure, with distinct biological pathways driving different health impacts between males and females [10, 11]. Physiologically, hormones play a critical role in central thermoregulation (temperature regulation by the brain and nervous system) and peripheral thermoregulation (heat loss through skin blood flow and sweating). Estrogen facilitates heat dissipation via vasodilation and lowers the sweating threshold, whereas progesterone contributes to heat conservation [12]. Therefore, these hormonal effects intrinsically influence water regulation and fluid balance, leading to increased susceptibility to high ambient temperatures in males, as they lack the protective effects of these hormones [12, 13].

However, current evidence on sex-specific heat vulnerability remains inconsistent. Several studies observed higher CVD risk from extreme heat in females [9, 14], while others reported higher risk in males [8, 15] or similar risks for both sexes [16]. These contradictory findings likely caused by complex interactions between biological mechanisms and varying geographical, social, and cultural factors across study populations. While the effects of extreme heat on cardiovascular health have been previously explored in various population subgroups [17–19], a comprehensive synthesis of sex-specific relative risks remains lacking.

In light of these considerations, this systematic review and meta-analysis aim to examine the sex-specific association between extreme heat exposure and cardiovascular disease outcomes. The focus of the analysis is synthesizing epidemiological evidence from the past decade in males and females and quantifying sex-specific relative risks of CVD responses to extreme heat exposure through meta-analysis.

## Methods

### Search methods

We conducted a comprehensive literature search in PubMed, Embase, and Scopus. The Preferred Reporting Items for Systematic Review and Meta-Analysis (PRISMA) guidelines were followed. YZ and LL developed the search strategy, which was subsequently refined following consultation with a university librarian. Key terms related to health outcomes included “cardiovascular disease”, “heart attack”, “heart failure”, and “cardiovascular mortality”. These were paired with exposure-related terms such as “extreme heat”, “heatwaves”, “temperature extremes”, and “high temperatures”. We also included terms specifically addressing sex differences, such as “sex”, “gender”, “male”, “female”, “men”, and “women”. We included peer-reviewed studies published in English from 2004, to 2024. A complete search was performed on Feb 10, 2024, and an updated search was performed on Nov 10, 2024, to capture recent publications. Additionally, reference lists of included articles and relevant reviews were screened to identify further studies.

### Selection criteria

We manually screened the abstracts of all studies identified with our search strategy. Studies were eligible if they reported on cardiovascular outcomes associated with heat exposure, and analyzed results stratified by sex. The analysis of included articles explored the following aspects: Extreme Heat definition: Two major heat exposure metrics: (1) frequency of heat wave days, which focused on counting the number of days that meet a specific threshold for extreme heat; (2) incremental temperature increase, which examined the health impact of each degree (1 °C) increase in temperature. Primary characteristics of the study design: Each article should cover main characteristics such as the specific cardiovascular outcomes analyzed, sources of medical data, geographic range of the study and Sex difference comparison. Quantification of extreme heat’s impact: The statistical methods utilized and the reported findings.

We excluded studies based on several criteria. Studies without estimation of an association between CVD related mortality/morbidity and heat were excluded. We also excluded studies that reported associations only for the entire population and did not report sex groups, as well as studies focusing solely on seasonal variations without specific consideration of extreme temperatures. Commentaries, editorials, and review articles were not included. Additionally, we excluded studies or vulnerability subgroups (within a study) with either no comparison group or no reference group; if a study assessed only one of the sex groups, it was not possible to assess heterogeneity, thus such estimates were not considered.

### Data extraction and quality assessment

Two reviewers (YZ and LL) independently screened titles, abstracts, and full texts using the Covidence platform [20]. Data extracted included author, year of publication, study location and period, methods of analysis, metrics of heat exposure, sex-specific outcomes, confounders, and main findings. To enable investigation of the possible effects of climate variability on heat-related cardiovascular risks, we added coordinates to each city or region-specific estimate using Google maps to determine the climate zone location following the Köppen-Geiger climate zones (a classification system that categorizes regions based on their climatic characteristics) [21].

The effect estimates for cardiovascular risks were obtained from published tables, figures, text descriptions and supplemental material. If quantity data was not provided in the original paper, we used WebPlotDigitizer (version 4.5) [22] to quantity and extract data from visualization images. Here we included relative risk (RR) estimates, or effect estimates that could be converted to RR (i.e., percentage change or excess risk, incidence rate ratio, and odds ratio). We converted these effect estimates to a standardised increment (1 °C) of high temperature, assuming a log-linear exposure-response relationship (i.e., a constant RR per unit increase in temperature above the defined reference temperature, which was reported in each study) [9]. For studies reporting percent changes, or ORs of health outcomes per unit increase in temperature, the effect estimates were converted to relative risks (RRs) using the equation: PC = (OR-1) × 100, and RR = OR/[(1-P0) + (P0 × OR)], where P0 = the incidence of the non-exposed group. We assumed RR = OR in this review [16]. In order to convert estimates of increases in health outcomes corresponding to 1 °C increase in temperature, or for studies reporting the effect of X degrees increase above a reference temperature point, the effect size was divided by X [16]. For studies reporting percentile-based RR estimates, we recorded the estimates per absolute change in temperature and calculated the log-RR, assuming a log-linear relationship in the range of temperature percentiles [23]. When studies did not identify or recommend a particular lag period, we extracted the highest mean RRs from either single or cumulative lag days [24]. This information was cross-verified, and discrepancies were resolved through discussion by the two reviewers and other co-authors.

We adhered to the Navigation Guide [25] for systematic reviews of observational studies in environmental health, involving a robust assessment of risk of bias (RoB), evidence quality across studies, and strength of the overall evidence. These assessments were conducted independently by the reviewers and discussed collectively to reach consensus. The study protocol was registered with PROSPERO (CRD42024547866).

### Statistical analysis

Due to the variation of the included studies designs and measurements, only papers assessing incremental temperature increase were analyzed in the meta-analysis. To quantify sex differences in heat-related cardiovascular risks, we employed the natural logarithm of the ratio of relative risks (RRR) between males and females. This approach, as described by Altman et al. [26] and Bassler et al. [27], allows for direct comparison of effect estimates between sexes within each study. We calculated the RRR by dividing the relative risk for males by the relative risk for females (RR_male / RR_female) and then taking the natural logarithm of this ratio. This method enables us to pool estimates across studies and provides a standardized measure of the magnitude and direction of sex differences in heat-related cardiovascular outcomes.

We conducted a random-effects meta-analysis using the DerSimonian-Laird method. Given the strong correlation between temperature metrics and their similar predictive ability, we pooled the estimates regardless of the exposure metrics. To assess whether there was a heterogeneous association for the observation, we conducted a Cochran Q test (in which 10% level was considered as significance) and categorised as low (≤ 25%), moderate (26–74%), or high (≥ 75%) using the I² statistic. Potential publication bias was assessed by Egger’s test and funnel plots [28]. Sensitivity analyses were conducted to test the robustness of the findings, including analyses by study design, risk of bias, and different temperature metrics. The heat wave studies were not assessed using the meta-analysis, while discussed through a narrative synthesis. This is because the study designs and methods for heat wave studies were not comparable to one another.

Further, meta-regressions to explain between-study heterogeneity were done considering study design (time series or case crossover), time period, latitude, annual mean temperature, and climate zones. Sensitivity analyses for the meta-analysis were done by separating studies by climate zones, study design (time series or case crossover), seasonality (warm season or whole year), and air pollution adjustment (yes or no). Statistical analyses were done using R statistical software (version 4.2–0).

## Results

**Fig. 1:**
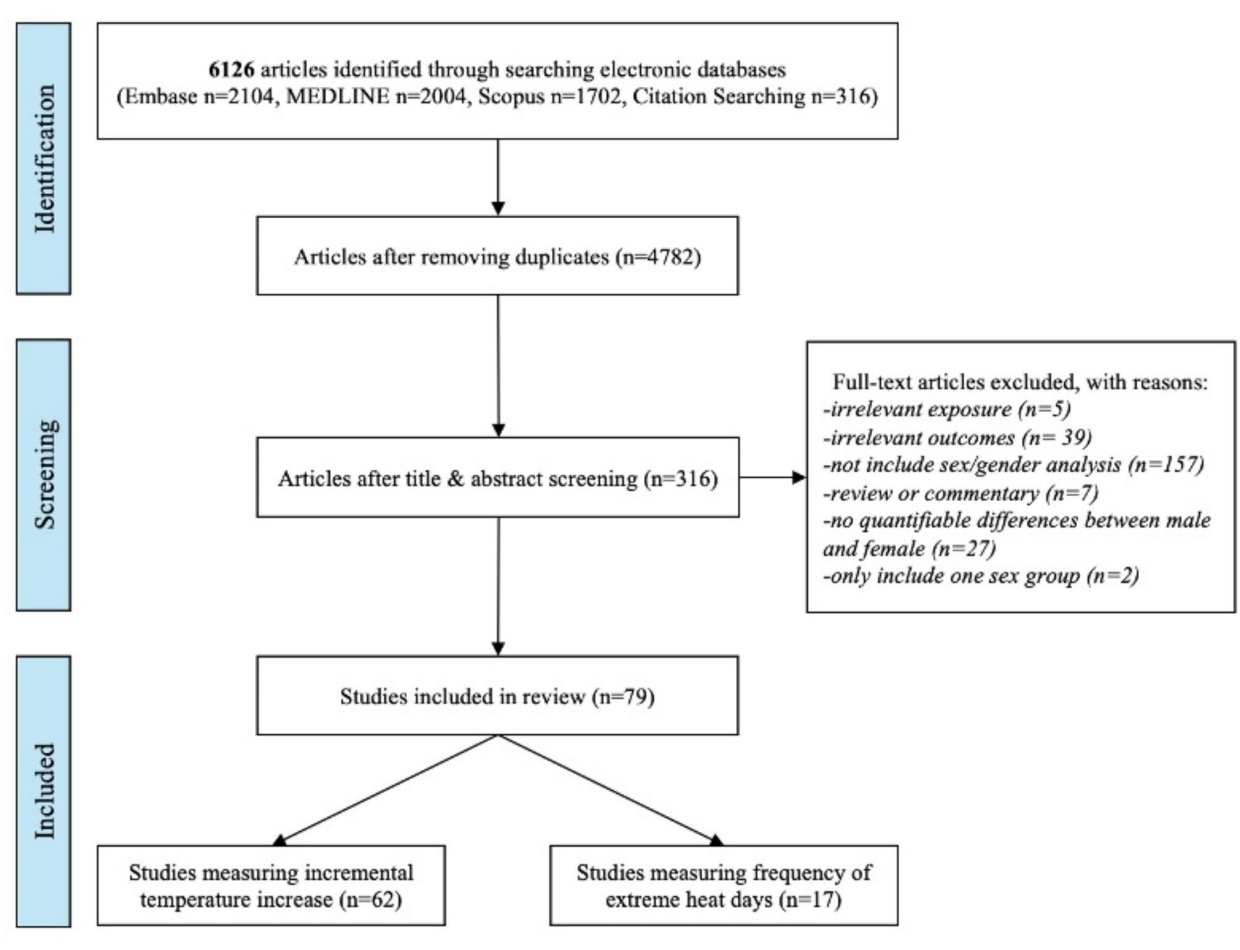
Flow chart

**Fig. 2:**
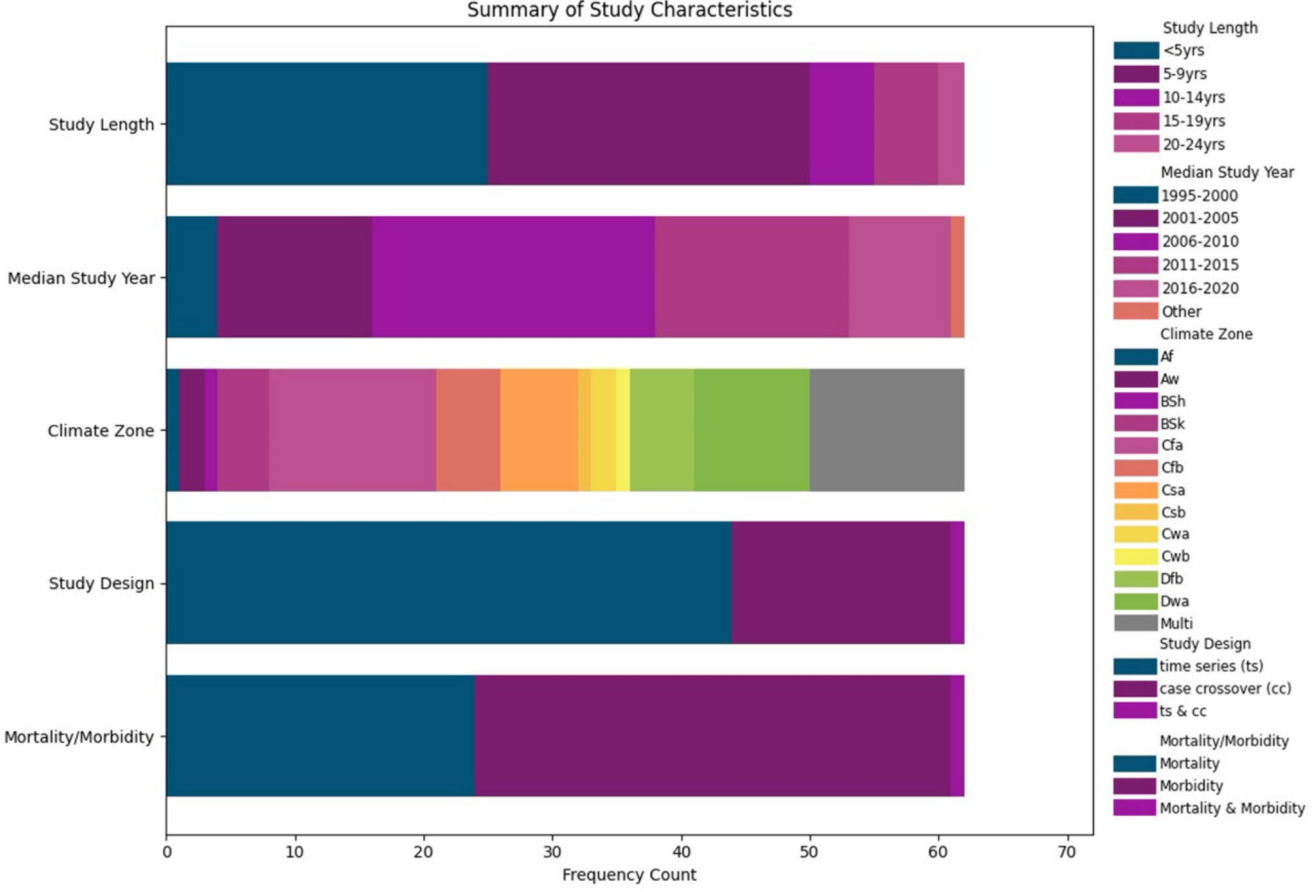
summary of characteristics for heat. Notes: - Median Study Year “Other” if two separate time periods. -Climate Zone based on Köppen-Geiger climate classification Af = Wet equatorial climate, Aw = Tropical wet-dry climate, BSh = Mid-latitude steppe and desert climate, BSk = Tropical and subtropical steppe climate, Cfa = Humid subtropical climate, Cfb = Marine west coast climate, Csa = Mediterranean climate, Csb = Mediterranean climate, Cwa = Humid subtropical climate, Cwb = Humid subtropical climate, Dfb = Humid continental climate, Dwa = Humid continental climate, Multi = multiple climate zones

Databases searches yielded 5810 studies, and 316 additional articles were identified through reference lists or the updated search. After removing duplicates and screening abstracts and titles, we reviewed 316 relevant studies for full-text eligibility assessment. Ultimately, we identified 79 studies that fulfilled the inclusion criteria for the systematic review (Fig. 1). Of these, 62 (78.5%) studies assessed high temperatures effects, while 17 (21.5%) studies examined heat wave effects. All the studies utilized a time-series (73% of total) or case-crossover (23%) design to assess the association between extreme heat exposure and cardiovascular mortality or morbidity (Fig. 2). All the studies were published between 2004 and 2024. Thirty-five studies were conducted in East Asia, 10 in Europe, five in North America, four in Oceania. From these total 79 articles, 41 observed the association between increasing temperature and higher risk of CVD outcomes in males while 41 articles in females as well (Appendix Table S2).

### Meta-analysis for high temperatures effects

**Fig. 3:**
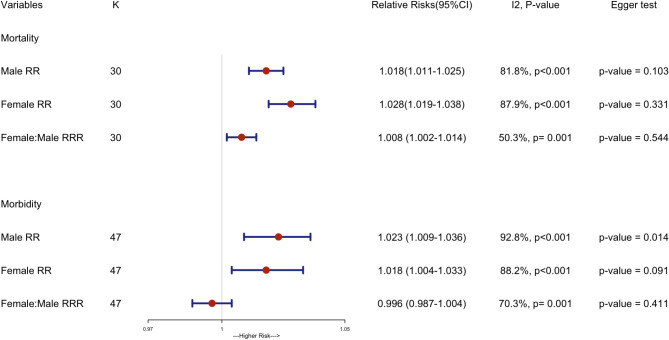
Findings from a random-effects meta-analysis showing change for cardiovascular disease mortality morbidity corresponding to a change per 1 °C increase in temperature. Notes: K:Number of estimates, RR: sex specific relative risk, RRR: pooled relative risk ratios

Of the 79 studies included in our review, 62 studies that assessed multiple cities/locations with 81 estimates were included into the meta-analysis. Analysis of pooled estimates showed that every 1 °C increase in temperature was significantly associated with a 1.8% increase in cardiovascular-related mortality in males (RR: 1.018 [95% CI: 1.011–1.025]) and a 2.8% increase in females (RR 1.028 [1.019–1.038]) while every 1 °C increase in temperature was significantly associated with a 2.3% increase in cardiovascular-related morbidity in males (RR: 1·023 [1·009–1·036]) and a 1.8% increase in females (RR: 1·018 [1.004–1.033]). Further, we found that the female: male pooled RRR was 1.008 (1.002, 1.014) for mortality, while 0.996 (0.987, 1.004) for morbidity (Fig. 3). These RRRs indicated that the relative risk for females is slightly higher than for males in the context of the association between heat and CVD related mortality, but not significant for morbidity. Significant heterogeneity was detected by I² (Mortality: I² = 46.6%, *p* = 0.003; Morbidity: I² = 67.2%, *p* < 0.0001), suggesting substantial variability in the effect estimates across studies.

The high heterogeneity (I^2^ > 50%) found in the pooled RRR suggests the existence of study characteristics influencing this variability. Of the study characteristics assessed, local temperature, climate zone, latitude or study period were not related to the heterogeneity in the pooled RRR in any of the meta-regression conducted. The pooled estimate for the ratios for females versus males remained similar after adjusting for the contrast definition. For mortality outcome, the pooled estimate was 1.011 (95% CI: 1.005, 1.017) while 1.002 (95% CI: 0.999, 1.005) for morbidity outcomes. The series of sensitivity analyses indicated consistency in the direction and magnitude of the association in the reviewed studies.

### Narrative synthesis of heat wave studies

Our review included 17 heat wave-related studies that were not suitable for meta-analysis due to methodological heterogeneity. These studies employed diverse definitions of heat waves, typically incorporating both temperature thresholds and duration criteria. Despite this variability, a consistent pattern emerged: heat waves were associated with increased risks of cardiovascular mortality and morbidity.

Although sex-specific impacts of heat waves on cardiovascular health were noted in multiple studies, findings were not consistent. For instance, Dong et al. [29] reported higher cardiovascular mortality risks for females and the elderly during heat waves in Beijing, China. Zacharias et al. [30] observed more pronounced effects of heat waves on females and individuals with chronic ischemic diseases in Germany. A comparative study in Europe [31] found that women in Rome were more heat-sensitive for CVD, while in Stockholm, men with chronic obstructive pulmonary disease (COPD) showed greater vulnerability. Several studies identified heightened vulnerability among specific demographic groups. The elderly (defined as age > 65 years in most papers) and females were frequently reported as being more susceptible to the adverse cardiovascular effects of heat waves [29, 30, 32, 33]. Some studies also did not detect any significant sex differences in the impact of heat waves on cardiovascular health. Wang et al. [34] found no substantial variation in cardiovascular mortality incidence rate ratios between sexes across three Australian cities. Similarly, Fisher et al. [35] reported mixed results for acute myocardial infarction hospitalization risks during extreme heat events among different demographic subgroups in Maryland, USA. These varied findings underscore the complexity of sex differences in heat wave vulnerability and suggest that other factors, such as local climate, socioeconomic conditions, and healthcare access, may interact with sex to influence cardiovascular outcomes during extreme heat events.

### Risk of bias assessment

**Fig. 4:**
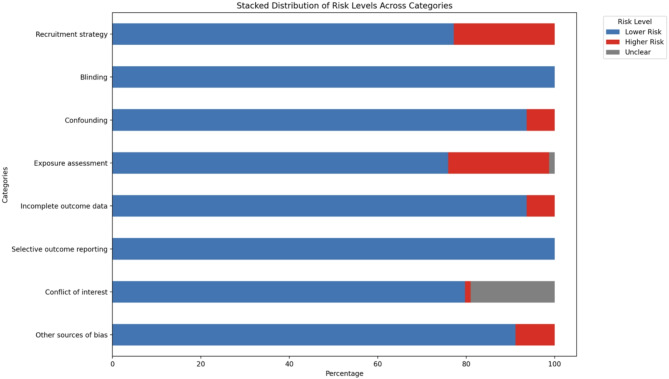
Weighted stacked bar plots indicating the percentage of the risk of bias judgements within each bias domain across reviewed studies of extreme heat exposure and cardiovascular disease events

The details of the RoB assessment criteria, and individual studies assessed for each domain, are in the supplement appendix (Appendix Table S4). In summary, we assessed the overall RoB of individual studies according to the key components of exposure, outcome, and confounding bias. Of the 79 included studies, the majority of included studies maintained good methodological quality across most domains (Fig. 4). Notable variation was observed that “Exposure assessment” with 74.7% of studies rated as low risk and 25.3% as higher risk. Common limitations in exposure assessment included lacking pollutants and meteorological variables. While some heterogeneity noted, sensitivity analyses excluding higher-risk studies did not substantially alter our primary findings.

## Discussion

In this systematic review, we assessed the published evidence supporting the presence of sex differences to heat-related mortality and morbidity. Using Cochran’s Q test we found evidence of particular vulnerability for females compared to males for mortality outcomes (pooled RRR: 1.008, 95% CI: 1.002, 1.014), but this difference was not significant for morbidity outcomes (pooled RRR: 0.996, 95% CI: 0.987, 1.004). However, substantial heterogeneity between studies (I² around 50%) complicates interpretation. While females may be at a marginally higher risk of heat-related cardiovascular mortality, vulnerability to extreme heat is not uniform across specific cardiovascular conditions or countries. These findings underscore the need for more sex-specific observations addressing the cardiovascular impacts of extreme heat exposure, while considering other individual and environmental risk factors.

Our findings observed a slightly higher heat-related cardiovascular mortality risk in females, highlighting the complexity of sex differences in heat vulnerability. This finding aligns with several large-scale studies included in our review. For instance, in the EuroHEAT study, the vulnerable subgroups identified when assessing the intensity and duration of heat-waves, consistently found higher mortality impacts among females compared to males [32], which is concordant with our results. Additional two European studies further supported this pattern, particularly noting increased susceptibility among elderly women [32, 36]. However, the literature presents a more complex picture, with some studies [37, 38] suggesting greater heat vulnerability in males. While several researchers have hypothesized that physiological mechanisms such as thermoregulatory and hormonal differences might explain sex-specific vulnerabilities [39, 40], the evidence for these biological explanations remains inconclusive.

This discrepancy highlights the need to consider more social related factors alongside sex in heat vulnerability assessments. Several previous reviews, such as Gronlund et al. [41], emphasized the role of sociocultural factors in determining heat exposure and adaptive behaviors. These studies suggested that community norms and gender roles can lead to different exposure levels and responses to heat between males and females. Occupational exposures, with males more likely to work in heat-exposed settings [42], and gender-based differences in health-seeking behaviors [43] may also contribute to the complex pattern of sex differences in heat vulnerability. Furthermore, the interaction between sex and socioeconomic factors was also evident in several studies [44, 45]. For example, Vandentorren et al. [46] who reported increased heat risk for unmarried men but not unmarried women during a heat wave in Paris, suggesting that social isolation may modify heat vulnerability differently by sex. The interplay of biological, social, and environmental factors appears to modulate the relationship between sex and heat vulnerability in ways that vary across populations and contexts. Future research should aim to disentangle these complex interactions to inform more targeted and effective heat adaptation strategies.

While our meta-analysis did not reveal significant sex differences for all cardiovascular outcomes, several studies have reported sex-specific variations in the impact of extreme heat on particular CVD manifestations. These results reflect the complexity of sex-based vulnerabilities to heat-related cardiovascular events. For ischemic heart disease (IHD) and ST-elevation myocardial infarction (STEMI), Bayentin et al. [47] and Gebhard et al. [48] observed a higher incidence of heat-related hospitalizations among younger females compared to age-matched males. This sex disparity is particularly noteworthy given that acute myocardial infarction (AMI) serves as the initial presentation of IHD in approximately 50% of cases, as reported in a previous cross-sectional study [49]. Physiological differences between sexes may contribute to these disparities. Women generally exhibit higher core temperatures, skin temperatures and heart rates compared to men, potentially reducing their heat tolerance. However, when controlling for body size and fat percentage, these thermoregulatory differences diminish, suggesting that physical characteristics may be more influential than sex alone in determining heat susceptibility [50]. Ha et al. [51] found that males were significantly more likely to be hospitalized during extreme heat events. This aligns with a systematic review on stroke epidemiology, which reported a 33% higher stroke incidence rate and a 41% higher stroke prevalence rate in men compared to women [52]. These differences may be attributed to the protective effect of estrogen against ischemic stroke in women, or to the higher prevalence of risk factors such as smoking and hypertension among men.

While our analysis focused on sex as an independent vulnerability factor, the complex interplay between sex and other demographic characteristics warrants careful consideration [9]. For example, age distribution differences between sexes could partially explain the heterogeneity in our findings. For instance, longer life expectancy for females mean a larger proportion of elderly females in many populations, potentially confounding the sex-specific heat vulnerability we observed [53, 54]. Additionally, age-related changes in cardiovascular function and thermoregulation may manifest differently between sexes. Other intersecting factors like socioeconomic status and comorbidities also likely modify sex-specific heat vulnerability [55]. Future research should employ interaction analyses to better understand how these factors collectively influence heat vulnerability, rather than treating them as independent modifiers.

The limited consideration of sex differences in extreme heat research represents an important gap that affects our understanding of heat-related health risks and the effectiveness of adaptation strategies. This issue is particularly concerning given the growing evidence of sex-specific responses to heat exposure. A comprehensive audit of heat adaptation research revealed that women accounted for only about 13% of the total participants in such studies [56]. This underrepresentation of females in heat-related research significantly limits the applicability of current guidelines for heat adaptation practices to the general female population. Even in studies that do include both sexes, there is often a failure to provide sex-stratified results. For example, in this review, 157 papers were excluded due to not including or providing sex-stratified results. This frequent omission of sex-stratified analysis represents a significant gap in our understanding of heat-related health risks and adaptation strategies.

## Strengths and limitations

This paper has several strengths. A key strength of this review is that it addresses the gaps in knowledge about the quantitative effects of extreme heat exposure on cardiovascular disease health outcomes in a specific aspect of sex difference. The studies have been assessed on the basis of best practice guidelines developed specifically for environmental health research. The PRISMA checklist was followed, and a search protocol was developed in collaboration with the research team and approved prior to initiating this review to ensure transparency and rigour in the methodology and to aid in replicability in the future.

However, there are a few limitations. We only considered peer-reviewed literature published in English which could lead to potential bias. Secondly, a significant limitation was the high heterogeneity among the included studies, which prevented the inclusion of all relevant research in our meta-analyses. This variability in study designs, exposure metrics, and outcome measures across the literature highlights the need for more standardized approaches in future research [57]. While most included studies (72 out of 79) adjusted for relative humidity in their statistical models, our analysis primarily focused on temperature effects and did not specifically examine how humidity might interact with or modify the sex-specific effects of heat on cardiovascular outcomes. Future research would benefit from exploring whether sex differences in heat vulnerability vary between humid and dry heat environments. Additionally, other study-related factors that were not assessed in this review, such as population age and sex structures, presence of local heat action plans or population’s resilience facing hot temperatures, could explain some of the residual heterogeneity. In addition, we did not assess the influence of lag effects on modification effects in this meta-analysis as well. Yet, mortality displacement could be heterogeneous because of subgroups in different populations. Further studies may address this matter. While many factors are highly correlated, in this review, we considered them independently as assessed in the majority of the studies. The intersectionality needs to be addressed in the future, for example, it is possible that sex differences in age distribution could explain some of this heterogeneity.

## Conclusion

This systematic review and meta-analysis suggest that females might be at an increased risk of heat-related cardiovascular mortality compared to males. The relationship between sex and extreme heat-related cardiovascular morbidity remains unclear, with inconsistent results across studies. The mechanisms underlying these differences are likely multifactorial, reflecting a complex interplay of biological, geographical, and sociocultural. Future research should aim to elucidate these pathways and inform the development of sex-specific interventions to mitigate the health impacts of extreme heat events. Incorporating sex-based considerations into climate change adaptation strategies will be crucial to ensuring that vulnerable populations are not overlooked in addressing this escalating public health threat.

## Electronic supplementary material

Below is the link to the electronic supplementary material.

Supplementary Material 1

## Data Availability

No datasets were generated or analysed during the current study.

## Abbreviations

CVD: Cardiovascular disease
RR: Relative risk
PRISMA: Preferred Reporting Items for Systematic Review and Meta-Analysis
OR: Odds ratio
RoB: Risk of bias
