# Supplementary material for: Sex differences in the impact of extreme heat on cardiovascular disease outcomes: a systematic review and meta-analysis
Source: Environ Health. 2025 Apr 12;24:20. doi: 10.1186/s12940-025-01175-6 (PMC11992858; doi:10.1186/s12940-025-01175-6)
Supplement: Supplementary file 1 — Supplementary Material 1 [file 12940_2025_1175_MOESM1_ESM.docx]

# Appendix

## **Title: Sex Differences in the Impact of Extreme Heat on Cardiovascular Disease Outcomes: A Systematic Review and Meta-Analysis**

Yusheng Zhou^1^, Léa Larochelle^2^, Fahima Afsari Khan^2^, Louise Pilote ^1,3^

Affiliations

1. Research Institute of McGill University Health Centre, McGill University, Montreal, Quebec, Canada
2. Faculty of Medicine and Health Sciences, McGill University, Montreal, Quebec, Canada
3. Division of General Internal Medicine, McGill University Health Center, Montreal, Quebec, Canada

Address for correspondence:

Louise Pilote MD MPH PhD

Centre for Outcomes Research and Evaluation

McGill University Health Centre

5252 de Maisonneuve West, 2B.39

Montréal QC H4A 3S5

louise.pilote@mcgill.ca

Tel: 514 934-1934 ext. 44722;

Fax: 514 843-1676

**List of Tables**

Table S1. PRISMA Checklist ……………………………………………………………………………………………………………………. 3

Table S2. Significant sex-specific findings for included studies ………………………………………………………………………………… 6

Table S3. Characteristics of included studies ……………………………………..……………………………………………………………… 8

Table S4. Judgement of overall risk of bias rating ………………………………..……………………………………………………………… 13

**List of Figures**

Figure S1. Forest plot shows summary findings for included studies investigating the association between high temperatures and cardiovascular disease mortality………………………………..………………………………………………………………………………………………… 17

Figure S2. Forest plot shows summary findings for included studies investigating the association between high temperatures and cardiovascular disease morbidity………………………………..……………………………………………………………………………………………….. 18

Figure S3. Funnel plots to explore publication bias………………………………..…………………………………………………………….. 19

Table S1. Preferred Reporting Items for Systematic Reviews and Meta-Analyses (PRISMA) checklist

| **Section and Topic** | **Item #** | **Checklist item** | **Location where item is reported** |
| --- | --- | --- | --- |
| **TITLE** | | |  |
| Title | 1 | Identify the report as a systematic review. | 1 |
| **ABSTRACT** | | |  |
| Abstract | 2 | See the PRISMA 2020 for Abstracts checklist. | Abstract |
| **INTRODUCTION** | | |  |
| Rationale | 3 | Describe the rationale for the review in the context of existing knowledge. | 2 |
| Objectives | 4 | Provide an explicit statement of the objective(s) or question(s) the review addresses. | 2 |
| **METHODS** | | |  |
| Eligibility criteria | 5 | Specify the inclusion and exclusion criteria for the review and how studies were grouped for the syntheses. | 3 |
| Information sources | 6 | Specify all databases, registers, websites, organisations, reference lists and other sources searched or consulted to identify studies. Specify the date when each source was last searched or consulted. | 2 |
| Search strategy | 7 | Present the full search strategies for all databases, registers and websites, including any filters and limits used. | 2-3 |
| Selection process | 8 | Specify the methods used to decide whether a study met the inclusion criteria of the review, including how many reviewers screened each record and each report retrieved, whether they worked independently, and if applicable, details of automation tools used in the process. | 3 |
| Data collection process | 9 | Specify the methods used to collect data from reports, including how many reviewers collected data from each report, whether they worked independently, any processes for obtaining or confirming data from study investigators, and if applicable, details of automation tools used in the process. | 4 |
| Data items | 10a | List and define all outcomes for which data were sought. Specify whether all results that were compatible with each outcome domain in each study were sought (e.g. for all measures, time points, analyses), and if not, the methods used to decide which results to collect. | 4 |
|  | 10b | List and define all other variables for which data were sought (e.g. participant and intervention characteristics, funding sources). Describe any assumptions made about any missing or unclear information. | 4 |
| Study risk of bias assessment | 11 | Specify the methods used to assess risk of bias in the included studies, including details of the tool(s) used, how many reviewers assessed each study and whether they worked independently, and if applicable, details of automation tools used in the process. | 5 |
| Effect measures | 12 | Specify for each outcome the effect measure(s) (e.g. risk ratio, mean difference) used in the synthesis or presentation of results. | 4-5 |
| Synthesis methods | 13a | Describe the processes used to decide which studies were eligible for each synthesis (e.g. tabulating the study intervention characteristics and comparing against the planned groups for each synthesis (item #5)). | 5-6 |
|  | 13b | Describe any methods required to prepare the data for presentation or synthesis, such as handling of missing summary statistics, or data conversions. | 6 |
|  | 13c | Describe any methods used to tabulate or visually display results of individual studies and syntheses. | 6 |
|  | 13d | Describe any methods used to synthesize results and provide a rationale for the choice(s). If meta-analysis was performed, describe the model(s), method(s) to identify the presence and extent of statistical heterogeneity, and software package(s) used. | 5-6 |
|  | 13e | Describe any methods used to explore possible causes of heterogeneity among study results (e.g. subgroup analysis, meta-regression). | 6 |
|  | 13f | Describe any sensitivity analyses conducted to assess robustness of the synthesized results. | 6 |
| Reporting bias assessment | 14 | Describe any methods used to assess risk of bias due to missing results in a synthesis (arising from reporting biases). | 6 |
| Certainty assessment | 15 | Describe any methods used to assess certainty (or confidence) in the body of evidence for an outcome. | 6 |
| **RESULTS** | | |  |
| Study selection | 16a | Describe the results of the search and selection process, from the number of records identified in the search to the number of studies included in the review, ideally using a flow diagram. | 6 |
|  | 16b | Cite studies that might appear to meet the inclusion criteria, but which were excluded, and explain why they were excluded. | 6 |
| Study characteristics | 17 | Cite each included study and present its characteristics. | 6-7; Appendix |
| Risk of bias in studies | 18 | Present assessments of risk of bias for each included study. | 9 |
| Results of individual studies | 19 | For all outcomes, present, for each study: (a) summary statistics for each group (where appropriate) and (b) an effect estimate and its precision (e.g. confidence/credible interval), ideally using structured tables or plots. | 7-9 |
| Results of syntheses | 20a | For each synthesis, briefly summarise the characteristics and risk of bias among contributing studies. | 7-9 |
|  | 20b | Present results of all statistical syntheses conducted. If meta-analysis was done, present for each the summary estimate and its precision (e.g. confidence/credible interval) and measures of statistical heterogeneity. If comparing groups, describe the direction of the effect. | 7-9 |
|  | 20c | Present results of all investigations of possible causes of heterogeneity among study results. | 7-9 |
|  | 20d | Present results of all sensitivity analyses conducted to assess the robustness of the synthesized results. | 7-9 |
| Reporting biases | 21 | Present assessments of risk of bias due to missing results (arising from reporting biases) for each synthesis assessed. | 9 |
| Certainty of evidence | 22 | Present assessments of certainty (or confidence) in the body of evidence for each outcome assessed. | 9 |
| **DISCUSSION** | | |  |
| Discussion | 23a | Provide a general interpretation of the results in the context of other evidence. | 9 |
|  | 23b | Discuss any limitations of the evidence included in the review. | 13 |
|  | 23c | Discuss any limitations of the review processes used. | 13 |
|  | 23d | Discuss implications of the results for practice, policy, and future research. | 14 |
| **OTHER INFORMATION** | | |  |
| Registration and protocol | 24a | Provide registration information for the review, including register name and registration number, or state that the review was not registered. | 5 |
|  | 24b | Indicate where the review protocol can be accessed, or state that a protocol was not prepared. | 5 |
|  | 24c | Describe and explain any amendments to information provided at registration or in the protocol. | 5 |
| Support | 25 | Describe sources of financial or non-financial support for the review, and the role of the funders or sponsors in the review. | 14 |
| Competing interests | 26 | Declare any competing interests of review authors. | 14 |
| Availability of data, code and other materials | 27 | Report which of the following are publicly available and where they can be found: template data collection forms; data extracted from included studies; data used for all analyses; analytic code; any other materials used in the review. | 4 |

Table S2. Significant findings for studies measuring heat and CVD disease outcomes including sex-stratified results

| **Heat Measurement** | **Climate zones*** | **Studies (N= Number of the total studies)** | **Number of observations found heterogeneous (Significant/Total)** |
| --- | --- | --- | --- |
| Ambient temperature studies | Group A (Tropical) | N= 2:  Yatim 2021(1), Burkart 2014(2). | Male(1/2); Female (1/2) |
|  | Group B (dry) | N=5:  García-Lledó 2020(3), Royé 2019(4),  Salvador 2023(5), Zhang 2018(6), Iranpour 2020(7). | Male( 2/5); Female (2/5) |
|  | Group C (mediterranean) ***-****Csa, Csb, Csc* | N=7  Vered 2020(8), Misailidou 2006(9),  Moghadamnia 2018(10), Vodonos 2017(11), Monteiro 2013(12), Kranc 2021(13), Moghadamnia 2018(14). | Male( 3/7); Female (4/7) |
|  | Group C (oceanic)  *-Cfa, Cfb* | N=18:  Cui 2019(15), Lin 2021(16), Lu 2020(17),  Zhan 2022(18), Xu 2021(19), Wichmann 2013(20), Yu 2021(21), Lam 2018(22), Wichmann 2011(23), Wichmann 2012(24), Ge 2018(25), Abrutzky 2012(26), Lu 2021(27), Saucy 2021(28), Xu 2023(29), Zhou 2017(30), Wichmann 2011(31), Huang 2014(32). | Male( 8/18); Female (8/18) |
|  | Group C (subtropical)  ***-****Cwa, Cwb* | N=3:  Zhai 2022(33), Ikefuti 2018(34), Li 2017(35). | Male( 2/3); Female (1/3) |
|  | Group D (continental) | N=14:  Li 2019(36), Ma 2020(37), Shen 2021(38), Lin 2009(39), Zhai 2022(40), Rowland 2020(41), Cho 2018(42), Li 2021(43), Liu 2018(44),  Han 2016(45), Gerhard 2018(46), Xing 2020(47), Tian 2012(48), Grjibovski 2013(49). | Male(6/14); Female (4/14) |
|  | Multiple countries | N=12:  Wang 2020(50), Cleland 2023(51), Jiang 2022(52), Lee 2014(53), Son 2014(54), Kwon 2015(55), Chen 2014(56), Lim 2013(57), Wang 2015(58), Ban 2017(59), Onozuka 2017(60), Basu 2008(61). | Male( 6/12); Female (8/12) |
| Heat Wave Studies | Group A (Tropical) | N=2:  Phung 2016(62), Phung 2017(63) | Male(1 /2 ); Female ( 1/2) |
|  | Group C (oceanic)  *- Cfa, Cfb* | N=7:  Campbell 2019(64), Ha 2014(65), Fisher 2017(66), Huang 2010(67), Zeng 2014(68), Zacharias 2014(69), Moraes 2022(70). | Male(5 /7 ); Female ( 6/7 ) |
|  | Group D (continental) | N=5:  Urban 2014(71), Dong 2016(72), Tian 2013(73), Percic 2018(74), Hanzlíková 2015(75) . | Male(4/5 ); Female (4/5 ) |
|  | Multiple countries | N=4:  Ponjoan 2017(76), Fonseca-Rodríguez 2021(77), Oudin Åström 2015(78), D'Ippoliti 2010(79). | Male(3/4 ); Female (2/4 ) |

Notes: -Climate Zone based on Köppen-Geiger climate classification;

Group A includes: Af= Wet equatorial climate, Aw= Tropical wet-dry climate;

Group B includes: BSh= Mid-latitude steppe and desert climate, BSk= Tropical and subtropical steppe climate;

Group C (oceanic) includes: Cfa= Humid subtropical climate, Cfb= Marine west coast climate;

Group C (mediterranean) includes: Csa= hot-summer Mediterranean climate, Csb= warm-summer Mediterranean climate;

Group C (subtropical) inclues: Cwa= monsoon-influenced humid subtropical climate, Cwb= subtropical highland climate or Monsoon-influenced temperate oceanic climate;

Group D includes: Dfb= hot-summer humid continental climate, Dwa= warm-summer humid continental climate;

Multi= multiple climate zones

Table S3. Characteristics of included studies

| Studies | Country catergory | country/city | Climate Zone | Study period | Study Design | Measure types | Outcome | Study period (Season) | Outcome measurment | Population (ages) | Air pollution adjustment |
| --- | --- | --- | --- | --- | --- | --- | --- | --- | --- | --- | --- |
| Campbell, 2019(64) | Oceania | Australia | Cfb | 2008-2016 | cc | Heat wave | ER presentation | December to February | CVD (I00-I99, G45-46) | All | PM2.5 |
| Ha, 2014(65) | North America | USA | Cfa | 1994-2000 | cc | Heat wave | First stroke hospitalizations | May-September | ICD (I430-438 except 4320–4321, 4329 and 4370–4371) | 65y+ | PM10, O3 |
| Ponjoan, 2017(76) | Europe | Spain | Multi | 2006-2013 | cc | Heat wave | Hospitalization | Annual | CVD (I00-I99) | All | PM10, O3, NO2, SO2, CO |
| Fonseca-Rodríguez , 2021(77) | Europe | Sweden | Multi | 1991-2014 | ts | Heat wave | Hospitalization | May-August | CVD (I00-I99) | All | - |
| Fisher, 2017(66) | North America | USA | Cfa | 2000-2012 | cc | Heat wave | Hospitalizations | June-August | AMI | 18y+ | - |
| Urban , 2014(71) | Europe | Czech Republic | Dfb | 1994-2009 | cs | Heat wave | Morbidity and Mortality | Annual | CVD (I00-I99) | All | - |
| Phung, 2017(63) | East Asia | Vietnam | Aw | 2002-2015 | ts | Heat wave | Morbidity/Hospitalization | Annual | CVD (I00-I99) | All | - |
| Huang, 2010(67) | East Asia | China,Shanghai | Cfa | 2003 | ts | Heat wave | Mortality | June-September | CVD (I00-I99) | All | - |
| Dong, 2016(72) | East Asia | China,Beijing | Dwa | 2006-2009 | ts | Heat wave | Mortality | June-August | CVD (I00-I99) | All | - |
| Tian, 2013(73) | East Asia | China,Beijing | Dwa | 2000-2011 | ts | Heat wave | Mortality | May-September | CHD (I120-I125) | All | - |
| Oudin Åström, 2015(78) | Europe | Europe | Multi | 2000-2008 | ts | Heat wave | Mortality | May-September | CVD (I00-I99) | 50y+ | - |
| D'Ippoliti, 2010(79) | Europe | Europe | Multi | 1990-2004 | ts | Heat wave | Mortality | June-August | CVD (I00-I99) | 65y+ | SO2, TSP, PM10, PM2.5, NO2, O3, CO |
| Percic, 2018(74) | Europe | Slovenia | Dfb | 2003 and 2015 | cs | Heat wave | Mortality | May-September | CVD (I00-I99) | 5y+ | - |
| Zeng, 2014(68) | East Asia | China,Guangdong | Cfa | 2006-2010 | ts | Heat wave | Mortality | May-September | CVD (I00-I99) | All | - |
| Hanzlíková, 2015(75) | Europe | Czech Republic | Dfb | 1994-2009 | ts | Heat wave | Mortality and morbidity | June-August | CVD (I00-I99) | All | - |
| Zacharias, 2014(69) | Europe | Germany | Cfb | 2001-2010 | cs | Heat wave | Mortality and morbidity | May–September | IHD (I20-I25) | All | - |
| Moraes, 2022(70) | South America | Brazil | Cfa | 2006-2015 | cs | heat wave | Mortality | Annual | CVD (I00-I99) | 65y+ | PM10 |
| Vered, 2020(8) | Middle east | Israel | Csa | 2014-2016 | cc | Ambient temperature | Admission | June-September | Stroke (I60-I69) | 50y+ | PM2.5 |
| Cui, 2019(15) | East Asia | China,Hefei | Cfa | 2015-2017 | ts | Ambient temperature | CVD hospitalization | Annual | CVD (I00-I99) | All | PM10, SO2, NO2 |
| Li, 2019(36) | North America | USA,New York | Dfb | 2005-2013 | cc | Ambient temperature | ED visits | April-October | CVD (I00-I99) | 65y+ | PM2.5 |
| Wang, 2020(50) | East Asia | China Multiple cities | Multi | 2014-2017 | ts | Ambient temperature | Emergency department visits (EDVs) | June-August | CVD (I00-I99) | All | PM2.5, PM10, O3, CO, NO2, SO2 |
| Ma, 2020(37) | East Asia | China,Beijing | Dwa | 2009-2012 | ts | Ambient temperature | ER visits | Annual | CVD (I00-I99) | 15y+ | PM10, PM2.5 |
| Lin, 2021(16) | East Asia | Taiwan 5 cities | Cfa | 2000-2014 | ts | Ambient temperature | ER visits (ERV) | Annual | IHD (ICD-9-CM 410-414 and ICD-10-CM I20-I25) | 40y+ | PM10, SO2, NO, O3 |
| Shen, 2021(38) | East Asia | China,Shenyang | Dwa | 2014-2017 | ts | Ambient temperature | Hospital admission | Annual | CVD (I00-I99) | All | - |
| Lin, 2009(39) | North America | USA New York | Dfb | 1991-2004 | ts | Ambient temperature | Hospital admission | June-August | CVD (I00-I99) | All | O3, PM10, PM2.5, NO2, CO, SO2 |
| Zhai, 2022(33) | East Asia | China,Pingliang | Dwa | 2014-2015 | ts | Ambient temperature | Hospital admissions | Annual | CVD (I00-I99) | Adult and elderly | - |
| Lu, 2020(17) | Oceania | Australia,Queensland | Cfa | 1995-2016 | cc | Ambient temperature | Hospitalisations | Annual | CVD (I00-I99) | All | - |
| Zhan , 2022(18) | East Asia | China,Fujian | Cfa | 2010-2016 | ts | Ambient temperature | Hospitalization | Annual | CVD (I00-I99) | All | PM10, NO2, CO, SO2 |
| Cleland, 2023(51) | North America | USA | Multi | 2000-2017 | ts | Ambient temperature | Hospitalization | Annual | CVD (I00-I99) | 65-114 y | - |
| Xu, 2021(19) | Oceania | Australia,Brisbane | Cfa | 2005-2013 | cc | Ambient temperature | Hospitalizations | Annual | Stroke (I60-I69) | All | NO2, PM10 |
| Wichmann, 2013(20) | Europe | Sweden | Cfb | 1985-2010 | cc | Ambient temperature | Hospitalizations | Annual | MI | 23-102 | PM10,NO2,O3 |
| Yu, 2021(21) | East Asia | China,Wuhan | Cfa | 2011-2018 | ts | Ambient temperature | Morbidity | Annual | Aortic dissection | All | SO2, NO2 |
| Jiang, 2022(52) | East Asia | China | Multi | 2015-2021 | cc | Ambient temperature | Morbidity | Annual | Acute myocardial infarction (AMI) | All | PM2.5, PM10, O3, NO2, SO2,CO |
| García-Lledó, 2020(3) | Europe | Spain | Bsk | 2013-2017 | ts | Ambient temperature | Morbidity | Annual | STEMI | All | - |
| Rowland, 2020(41) | North America | USA,NY | Dfb | 2000-2015 | cc | Ambient temperature | Primary MI hospital admissions | Annual | MI (ICD9 and 10) | All | - |
| Lam, 2018(22) | East Asia | China,Hongkong | Cfa | 2002-2011 | ts | Ambient temperature | AMI admissions | Annual | AMI (ICD-9 = 410.00–410.99) | All | PM10, SO2, NO2, O3 |
| Cho, 2018(42) | East Asia | South Korea | Dwa | 2005-2009 | ts | Ambient temperature | ED visits | Annual | Stroke (I60-I69) | 40y+ | O3, PM2.5 |
| Wichmann, 2011(31) | Europe | Denmark | Cfb | 2002-2006 | cc | Ambient temperature | Emergency hospital admissions | Annual | CVD (I00-I99) | 19y+ | PM10, NO2, CO |
| Lee, 2014(53) | East Asia | South Korea | Multi | 2006-2010 | ts | Ambient temperature | Emergency visits | Annual | ACS | All | PM10, NO2, SO2, ozone, and CO |
| Misailidou, 2006(9) | Europe | Greece | Csa | 2003-2004 | ts | Ambient temperature | ER admission/Morbidity | Annual | ACS | All | - |
| Son, 2014(54) | East Asia | South Korea | Multi | 2003-2008 | ts | Ambient temperature | ER admissions/Morbidity | Warm season (March-August) | CVD (I00-I99) | All | - |
| Li, 2021(43) | East Asia | China,Beijing | Dwa | 2017-2019 | ts | Ambient temperature | Hospital admission | Annual | ACS | All | PM10, NO2, CO, SO2, O3, PM2.5 |
| Wichmann, 2012(24) | Europe | Denmark | Cfb | 1999-2006 | cc | Ambient temperature | Hospital admission | April-September | AMI (I21-I22) | 19y+ | PM10, NO2 and CO |
| Moghadamnia, 2018(14) | Middle east | Iran | Csa | 2005-2014 | ts | Ambient temperature | Hospital admission | Annual | ACS | All | - |
| Vodonos, 2017(11) | Middle east | Israel | Csa | 2012-2014 | cc | Ambient temperature | Hospital admission | Annual | Stroke (I60-I69) | All | - |
| Kwon, 2015(55) | East Asia | South Korea | Multi | 2004-2012 | ts | Ambient temperature | Hospital admission | June-August | AMI (I210-214 and 219) | 20y+ | PM10, NO2, SO2, CO, O3 |
| Liu, 2018(44) | East Asia | China,Beijing | Dwa | 2013-2016 | ts | Ambient temperature | Hospital admissions | Annual | CVD (I00-I99) | All | PM10, O3, NO2, SO2 |
| Phung, 2016(62) | East Asia | Vietnam | Aw | 2004-2013 | ts | Ambient temperature | Hospital admissions | Annual | CVD (I00-I99) | All | - |
| Ge , 2018(25) | East Asia | China,Shanghai | Cfa | 2013-2015 | ts | Ambient temperature | Hospital admissions for RHD | Annual | ICD (I05-I09) | All | PM2.5, O3 |
| Royé, 2019(4) | Europe | Spain | Bsk | 2001-2013 | ts | Ambient temperature | IS Hospitalization and Mortality | Annual | ICD9 (I433-435); ICD10 (163-164) | 15y+ | NO2, O3, SO2, PM10 |
| Han, 2016(45) | East Asia | South Korea | Dwa | 2004-2014 | ts | Ambient temperature | Morbidity | Annual | Stroke (I60-I69) | 19y+ | PM10, NO2, O3 |
| Salvador, 2023(5) | Europe | Spain | Bsk | 2015-2018 | cc | Ambient temperature | Morbidity | June-September | Acute cardiovascular event (CVE) | 40-75 | PM10, NO2, O3 |
| Monteiro, 2013(12) | Europe | Portugal | Csb | 2006 | ts | Ambient temperature | Morbidity and mortality | Annual | CVD (I00-I99) | All | PM10, O3 |
| Gerhard, 2018(46) | North America | Canada,Montreal | Dfb | 2010-2014 | ts | Ambient temperature | Morbidity/ER admission | Annual | STEMI | All | - |
| Kranc, 2021(13) | Middle east | Israel | Csa | 2016-2017 | cc | Ambient temperature | Emergency medical services (EMS) for Out-of-hospital-cardiac arrest (OHCA) | May-August | OHCA | 18y+ | - |
| Abrutzky, 2012(26) | South America | Argentina | Cfa | 2002-2006 | ts | Ambient temperature | Mortality | Annual | CVD (I00-I99) | All | CO |
| Lu, 2021(27) | Oceania | Australia | Cfa | 1997-2013 | cc | Ambient temperature | Mortality | Annual | CVD (I00-I99) | All | - |
| Zhang, 2018(6) | East Asia | China,Yinchuan | Bsk | 2010-2015 | ts | Ambient temperature | Mortality | Annual | CVD (I00-I99) | All | - |
| Xing, 2020(47) | East Asia | China,Beijing | Dwa | 2006-2011 | ts | Ambient temperature | Mortality | Annual | CVD (I00-I99) | All | NO2, O3, SO2, PM2.5 |
| Zhai, 2022(40) | East Asia | China, Qingdao | Cwa | 2009-2017 | ts | Ambient temperature | Mortality | Annual | CVD (I00-I99) | All | - |
| Tian, 2012(48) | East Asia | China,Beijing | Dwa | 2000-2011 | ts and cc | Ambient temperature | Mortality | Annual | CHD (I120-I125) | All | - |
| Chen, 2014(56) | East Asia | China | Multi | 2009-2011 | ts | Ambient temperature | Mortality | Annual | Out-of-hospital coronary death (OHCD) | All | PM10, SO2, NO2 |
| Iranpour, 2020(7) | Middle east | Iran | Bsh | 2014-2018 | ts | Ambient temperature | Mortality | Annual | CVD (I00-I99) | All | PM2.5, PM10, NO2, CO, SO2 |
| Yatim, 2021(1) | East Asia | Malaysia | Af | 2006-2015 | ts | Ambient temperature | Mortality | Annual | CVD (I00-I99) | All | PM10, O3 |
| Saucy, 2021(28) | Europe | Switzerland | Cfb | 2000-2015 | cc | Ambient temperature | Mortality | Annual | CVD (I00-I99) | All | PM2.5, NO2 |
| Xu, 2023(29) | East Asia | China,Jiangsu | Cfa | 2015-2020 | cc | Ambient temperature | Mortality | Annual | MI | All | PM2.5, SO2, NO2, CO, and O3 |
| Lim, 2013(57) | East Asia | South Korea | Multi | 1992-2007 | ts | Ambient temperature | Mortality from ischemic and hemorrhagic strokes | Annual | (ICD 9, 430–432 and 433-436); (ICD10,I60-I62 and I63-I66) | All | PM10, O3 |
| Wang, 2015(58) | Oceania | Australia | Multi | 1988-2011 | ts | Ambient temperature | Mortality | Annual (different study periods) | - | All | - |
| Ikefuti, 2018(34) | East Asia | Brazil | Cwb | 2002-2011 | ts | Ambient temperature | Mortality | Annual | Stroke (I60-I69) | All | PM10, O3, SO2, NO2 |
| Ban, 2017(59) | East Asia | China | Multi | 2013-2015 | ts | Ambient temperature | Mortality | Annual | CVD (I00-I99) | All | O3 and PM2.5 |
| Li, 2017(35) | East Asia | China,Jinan | Cwa | 2007-2013 | ts | Ambient temperature | Mortality | May-September | CVD (I00-I99) | Alll | - |
| Zhou, 2017(30) | East Asia | China,Jiangsu | Cfa | 2009-2013 | ts | Ambient temperature | Mortality | May-September | CVD (I00-I99) | All | PM2.5, PM10, O3, NO2 |
| Wichmann, 2011(23) | Europe | Denmark | Cfb | 1999-2006 | cc | Ambient temperature | Mortality | Annual | CVD (I00-I99) | All | PM10, NO2, CO |
| Onozuka, 2017(60) | East Asia | Japan | Multi | 2005-2014 | ts | Ambient temperature | Mortality | Annual | Out-of-hospital cardiac arrest (OHCA) | 18y+ | - |
| Basu, 2008(61) | North America | USA | Multi | 1999–2003 | cc | Ambient temperature | Mortality | May-September | CVD (I00-I99) | All | - |
| Burkart, 2014(2) | East Asia | Bangladesh | Aw | 2002-2007 | ts | Ambient temperature | Mortality | March-May | CVD (I00-I99) | All | - |
| Moghadamnia, 2018(10) | Middle east | Iran | Csa | 2005-2014 | ts | Ambient temperature | Mortality | Annual | CVD (I00-I99) | A | - |
| Grjibovski , 2013(49) | Middle east | Kazakhstan | Dfb | 2000-2001; 2006-2010 | ts | Ambient temperature | Mortality | Annual | CVD (I00-I99) | 18y+ | - |
| Huang, 2014(32) | East Asia | China | Cfa | 2008-2011 | ts | Ambient temperature | Mortalty | June - August; December - February | CVD (I00-I79) | All | PM2.5, PM10, SO2, NO |

Table S4. Judgement of overall risk of bias rating

| **Studies** | **Heat measurment** | **Risk of bias** | | | | | | | |
| --- | --- | --- | --- | --- | --- | --- | --- | --- | --- |
|  |  | **Recruitment strategy** | **Blinding** | **Confounding** | **Exposure assessment** | **Incomplete outcome data** | **Selective outcome reporting** | **Conflict of interest** | **Other sources of bias** |
| Abrutzky,2012 | High Temp | 1 | 1 | 1 | 2 | 1 | 1 | 3 | 1 |
| Ban,2017 | High Temp | 1 | 1 | 1 | 1 | 1 | 1 | 1 | 1 |
| Basu,2008 | High Temp | 1 | 1 | 1 | 2 | 1 | 1 | 1 | 1 |
| Burkart,2014 | High Temp | 2 | 1 | 1 | 2 | 1 | 1 | 3 | 1 |
| Campbell,2019 | Heat Wave | 1 | 1 | 1 | 1 | 1 | 1 | 1 | 1 |
| Chen,2014 | High Temp | 1 | 1 | 1 | 1 | 1 | 1 | 1 | 1 |
| Cho,2018 | High Temp | 1 | 1 | 1 | 1 | 1 | 1 | 1 | 1 |
| Cleland,2023 | High Temp | 2 | 1 | 1 | 2 | 1 | 1 | 1 | 1 |
| Cui,2019 | High Temp | 2 | 1 | 1 | 1 | 1 | 1 | 1 | 1 |
| D'Ippoliti,2010 | Heat Wave | 1 | 1 | 1 | 1 | 1 | 1 | 1 | 1 |
| Dong,2016 | Heat Wave | 1 | 1 | 1 | 1 | 1 | 1 | 1 | 1 |
| Fisher,2017 | Heat Wave | 2 | 1 | 1 | 1 | 1 | 1 | 1 | 2 |
| Fonseca-Rodríguez,2021 | Heat Wave | 1 | 1 | 1 | 2 | 1 | 1 | 1 | 2 |
| García-Lledó,2020 | High Temp | 2 | 1 | 1 | 2 | 1 | 1 | 1 | 1 |
| Ge,2018 | High Temp | 1 | 1 | 1 | 1 | 1 | 1 | 1 | 1 |
| Gebhard,2018 | High Temp | 2 | 1 | 1 | 1 | 1 | 1 | 1 | 1 |
| Grjibovski,2013 | High Temp | 1 | 1 | 1 | 1 | 1 | 1 | 1 | 2 |
| Ha,2014 | Heat Wave | 2 | 1 | 1 | 1 | 1 | 1 | 1 | 1 |
| Han,2016 | High Temp | 1 | 1 | 1 | 1 | 1 | 1 | 1 | 1 |
| Hanzlíková,2015 | Heat Wave | 1 | 1 | 1 | 1 | 1 | 1 | 3 | 2 |
| Huang (Jixia),2014 | High Temp | 2 | 1 | 1 | 1 | 1 | 1 | 1 | 1 |
| Huang (Wei),2010 | Heat Wave | 1 | 1 | 1 | 1 | 1 | 1 | 3 | 1 |
| Ikefuti,2018 | High Temp | 1 | 1 | 1 | 2 | 1 | 1 | 1 | 2 |
| Iranpour,2020 | High Temp | 1 | 1 | 1 | 1 | 1 | 1 | 1 | 1 |
| Jiang,2022 | High Temp | 1 | 1 | 1 | 1 | 1 | 1 | 1 | 1 |
| Kranc,2021 | High Temp | 1 | 1 | 1 | 2 | 1 | 1 | 1 | 1 |
| Kwon,2015 | High Temp | 1 | 1 | 1 | 1 | 1 | 1 | 1 | 1 |
| Lam,2018 | High Temp | 1 | 1 | 1 | 1 | 1 | 1 | 1 | 1 |
| Lee,2014 | High Temp | 1 | 1 | 1 | 1 | 2 | 1 | 1 | 1 |
| Li (Jing),2017 | High Temp | 1 | 1 | 1 | 1 | 1 | 1 | 1 | 1 |
| Li (Mengxuan),2019 | High Temp | 1 | 1 | 1 | 2 | 1 | 1 | 1 | 1 |
| Li (Na),2021 | High Temp | 1 | 1 | 1 | 1 | 1 | 1 | 1 | 1 |
| Lim,2013 | High Temp | 1 | 1 | 1 | 1 | 1 | 1 | 1 | 1 |
| Lin (Yu-Kai),2021 | High Temp | 1 | 1 | 1 | 1 | 1 | 1 | 1 | 1 |
| Lin (Shao),2009 | High Temp | 1 | 1 | 1 | 1 | 1 | 1 | 3 | 1 |
| Liu,2018 | High Temp | 1 | 1 | 1 | 1 | 1 | 1 | 1 | 1 |
| Lu^1,2020 | High Temp | 1 | 1 | 1 | 1 | 1 | 1 | 3 | 1 |
| Lu^2,2021 | High Temp | 1 | 1 | 1 | 1 | 1 | 1 | 3 | 1 |
| Ma,2020 | High Temp | 1 | 1 | 1 | 1 | 1 | 1 | 1 | 1 |
| Misailidou,2006 | High Temp | 1 | 1 | 1 | 3 | 1 | 1 | 3 | 1 |
| Moghadamnia^1,2018 | High Temp | 1 | 1 | 1 | 1 | 1 | 1 | 1 | 1 |
| Moghadamnia^2,2018 | High Temp | 1 | 1 | 1 | 1 | 1 | 1 | 1 | 1 |
| Monteiro,2013 | High Temp | 1 | 1 | 1 | 1 | 2 | 1 | 1 | 1 |
| Moraes,2022 | Heat Wave | 1 | 1 | 1 | 2 | 1 | 1 | 1 | 1 |
| Onozuka,2017 | High Temp | 1 | 1 | 1 | 1 | 1 | 1 | 1 | 2 |
| Oudin Åström,2015 | Heat Wave | 1 | 1 | 1 | 2 | 1 | 1 | 1 | 1 |
| Percic,2018 | Heat Wave | 2 | 1 | 2 | 1 | 1 | 1 | 1 | 1 |
| Phung^1,2016 | High Temp | 2 | 1 | 1 | 1 | 1 | 1 | 1 | 1 |
| Phung^2,2017 | Heat Wave | 2 | 1 | 2 | 1 | 2 | 1 | 3 | 1 |
| Ponjoan,2017 | Heat Wave | 1 | 1 | 1 | 1 | 2 | 1 | 1 | 1 |
| Rowland,2020 | High Temp | 1 | 1 | 1 | 1 | 1 | 1 | 1 | 1 |
| Royé,2019 | High Temp | 1 | 1 | 1 | 1 | 1 | 1 | 1 | 1 |
| Salvador,2023 | High Temp | 2 | 1 | 1 | 2 | 1 | 1 | 1 | 1 |
| Saucy,2021 | High Temp | 2 | 1 | 1 | 1 | 1 | 1 | 1 | 1 |
| Shen,2021 | High Temp | 1 | 1 | 1 | 1 | 1 | 1 | 1 | 1 |
| Son,2014 | High Temp | 1 | 1 | 1 | 1 | 1 | 1 | 1 | 1 |
| Tian^1,2012 | High Temp | 1 | 1 | 1 | 1 | 1 | 1 | 1 | 1 |
| Tian^2,2013 | Heat Wave | 1 | 1 | 1 | 1 | 1 | 1 | 1 | 1 |
| Urban,2014 | Heat Wave | 1 | 1 | 1 | 1 | 1 | 1 | 3 | 1 |
| Vered,2020 | High Temp | 2 | 1 | 1 | 2 | 1 | 1 | 1 | 1 |
| Vodonos,2017 | High Temp | 1 | 1 | 1 | 1 | 1 | 1 | 3 | 2 |
| Wang (Xiao Yu),2015 | High Temp | 1 | 1 | 1 | 1 | 2 | 1 | 2 | 1 |
| Wang (Yu),2020 | High Temp | 2 | 1 | 1 | 1 | 1 | 1 | 1 | 1 |
| Wichmann^1,2011 | High Temp | 1 | 1 | 1 | 2 | 1 | 1 | 1 | 1 |
| Wichmann^2,2011 | High Temp | 1 | 1 | 1 | 2 | 1 | 1 | 1 | 1 |
| Wichmann^3,2012 | High Temp | 1 | 1 | 1 | 2 | 1 | 1 | 1 | 1 |
| Wichmann^4,2013 | High Temp | 1 | 1 | 1 | 2 | 1 | 1 | 1 | 1 |
| Xu (Zhiwei),2021 | High Temp | 1 | 1 | 1 | 1 | 1 | 1 | 1 | 1 |
| Xu^2 (Ruijun),2023 | High Temp | 1 | 1 | 1 | 2 | 1 | 1 | 1 | 1 |
| Yatim,2021 | High Temp | 1 | 1 | 1 | 1 | 1 | 1 | 1 | 1 |
| Yu,2021 | High Temp | 2 | 1 | 1 | 1 | 1 | 1 | 3 | 1 |
| Zacharias,2014 | Heat Wave | 1 | 1 | 1 | 1 | 1 | 1 | 1 | 1 |
| Zeng,2014 | Heat Wave | 1 | 1 | 2 | 1 | 1 | 1 | 1 | 1 |
| Zhai (Guanyu),2022 | High Temp | 1 | 1 | 2 | 1 | 1 | 1 | 3 | 1 |
| Zhai (Long),2022 | High Temp | 1 | 1 | 1 | 1 | 1 | 1 | 3 | 1 |
| Zhan,2022 | High Temp | 1 | 1 | 1 | 1 | 1 | 1 | 1 | 1 |
| Zhang,2018 | High Temp | 2 | 1 | 2 | 1 | 1 | 1 | 3 | 1 |
| Zhou,2017 | High Temp | 2 | 1 | 1 | 1 | 1 | 1 | 1 | 1 |
| Zing,2020 | High Temp | 1 | 1 | 1 | 1 | 1 | 1 | 1 | 1 |

Figure S1. Meta-analysis of the ratio of the relative ratios (RRR) according to sex (RRfemales/RRmales) for mortality outcomes; n=30 observations


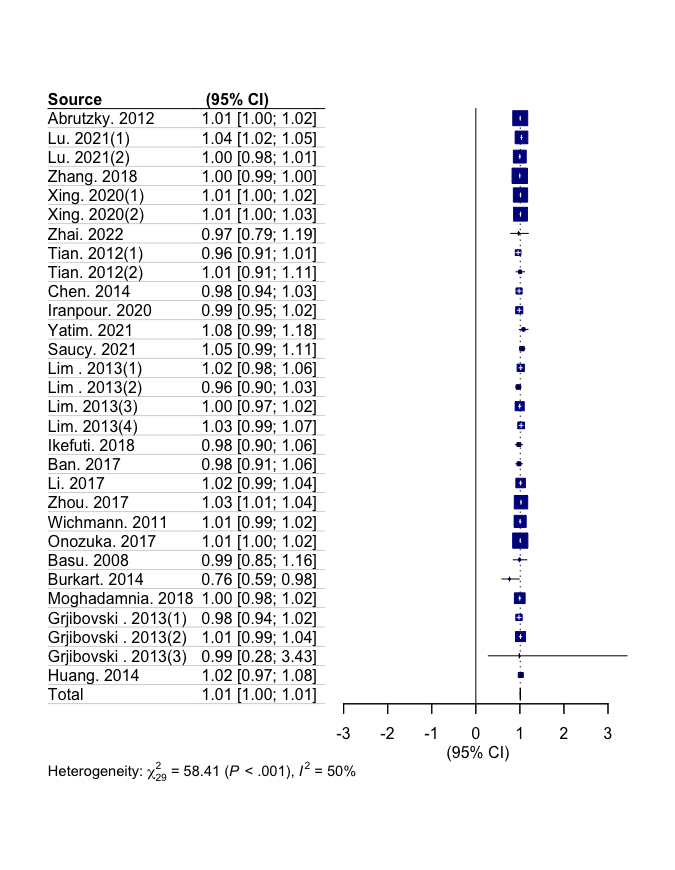


Figure S2. Meta-analysis of the ratio of the relative ratios (RRR) according to sex (RRfemales/RRmales) for morbidity outcomes; n=47 observations


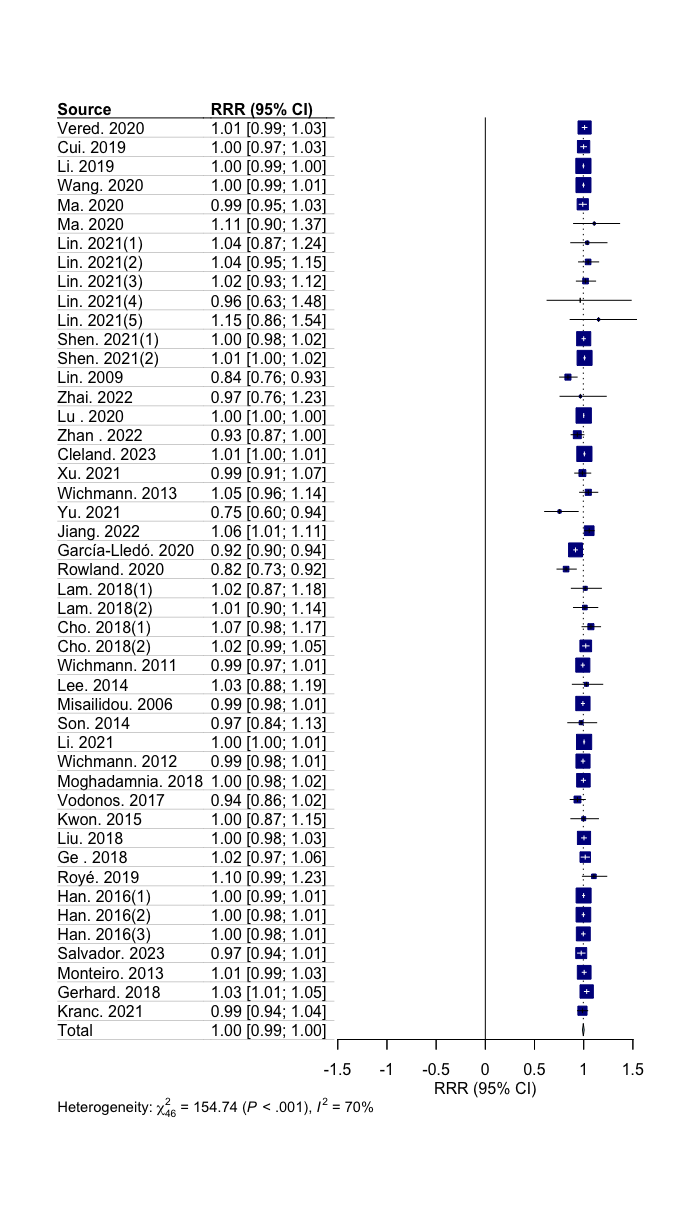


Figure S3. Funnel plots to explore publication bias


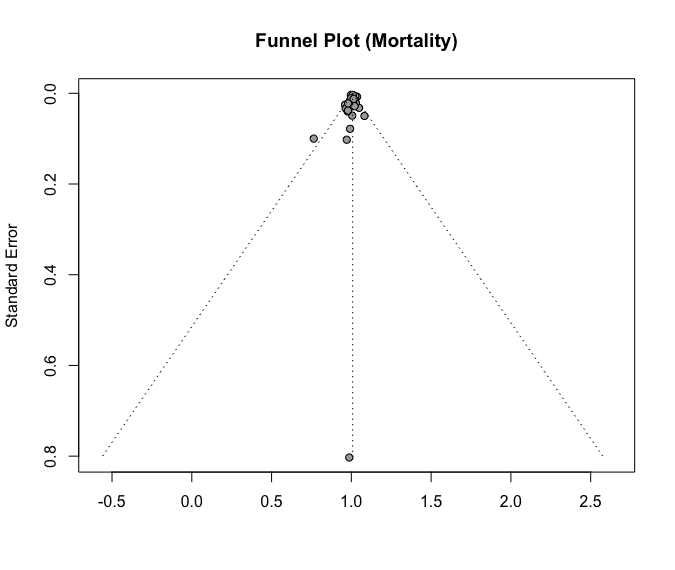

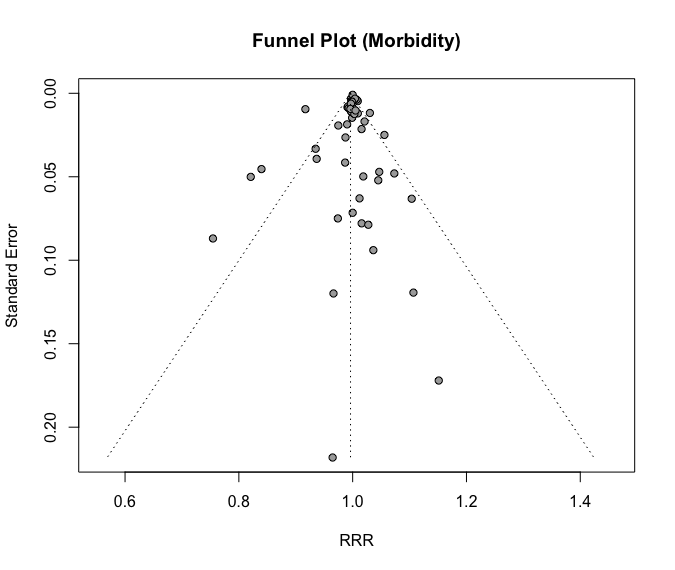


Refererence

1. Yatim ANM, Latif MT, Sofwan NM, Ahamad F, Khan MF, Mahiyuddin WRW, et al. The association between temperature and cause-specific mortality in the Klang Valley, Malaysia. Environ Sci Pollut Res Int. 2021 Nov;28(42):60209–20.

2. Burkart K, Breitner S, Schneider A, Khan MMH, Krämer A, Endlicher W. An analysis of heat effects in different subpopulations of Bangladesh. Int J Biometeorol. 2014 Mar;58(2):227–37.

3. García-Lledó A, Rodríguez-Martín S, Tobías A, Alonso-Martín J, Ansede-Cascudo JC, de Abajo FJ. Olas de calor, temperatura ambiente y riesgo de infarto de miocardio: un estudio ecológico en la Comunidad de Madrid. Rev Esp Cardiol. 2020 Apr 1;73(4):300–6.

4. Royé D, Zarrabeitia MT, Riancho J, Santurtún A. A time series analysis of the relationship between apparent temperature, air pollutants and ischemic stroke in Madrid, Spain. Environ Res. 2019 Jun 1;173:349–58.

5. Salvador C, Gullón P, Franco M, Vicedo-Cabrera AM. Heat-related first cardiovascular event incidence in the city of Madrid (Spain): Vulnerability assessment by demographic, socioeconomic, and health indicators. Environ Res. 2023 Jun 1;226:115698.

6. Zhang H, Wang Q, Zhang Y, Yang Y, Zhao Y, Sang J, et al. Modeling the impacts of ambient temperatures on cardiovascular mortality in Yinchuan: evidence from a northwestern city of China. Environ Sci Pollut Res. 2018 Feb 1;25(6):6036–43.

7. Iranpour S, Khodakarim S, Shahsavani A, Khosravi A, Etemad K. Modification of the effect of ambient air temperature on cardiovascular and respiratory mortality by air pollution in Ahvaz, Iran. Epidemiol Health. 2020;42:e2020053.

8. Vered S, Paz S, Negev M, Tanne D, Zucker I, Weinstein G. High ambient temperature in summer and risk of stroke or transient ischemic attack: A national study in Israel. Environ Res. 2020 Aug;187:109678.

9. Misailidou M, Pitsavos C, Panagiotakos DB, Chrysohoou C, Stefanadis C. Short-term effects of atmospheric temperature and humidity on morbidity from acute coronary syndromes in free of air pollution rural Greece. Eur J Cardiovasc Prev Rehabil. 2006 Oct 1;13(5):846–8.

10. Moghadamnia MT, Ardalan A, Mesdaghinia A, Naddafi K, Yekaninejad MS. The Effects of Apparent Temperature on Cardiovascular Mortality Using a Distributed Lag Nonlinear Model Analysis: 2005 to 2014. Asia Pac J Public Health. 2018 May;30(4):361–8.

11. Vodonos A, Novack V, Horev A, Abu Salameh I, Lotan Y, Ifergane G. Do Gender and Season Modify the Triggering Effect of Ambient Temperature on Ischemic Stroke? Womens Health Issues Off Publ Jacobs Inst Womens Health. 2017;27(2):245–51.

12. Monteiro A, Carvalho V, Oliveira T, Sousa C. Excess mortality and morbidity during the July 2006 heat wave in Porto, Portugal. Int J Biometeorol. 2013 Jan 1;57(1):155–67.

13. Kranc H, Novack V, Shtein A, Sherman R, Novack L. Extreme temperature and out-of-hospital-cardiac-arrest. Nationwide study in a hot climate country. Environ Health. 2021 Apr 5;20(1):38.

14. Moghadamnia MT, Ardalan A, Mesdaghinia A, Naddafi K, Yekaninejad MS. Association between apparent temperature and acute coronary syndrome admission in Rasht, Iran. Heart Asia. 2018 Oct 22;10(2):e011068.

15. Cui L, Geng X, Ding T, Tang J, Xu J, Zhai J. Impact of ambient temperature on hospital admissions for cardiovascular disease in Hefei City, China. Int J Biometeorol. 2019 Jun;63(6):723–34.

16. Lin YK, Zafirah Y, Ke MT, Andhikaputra G, Wang YC. The effects of extreme temperatures on emergency room visits-a population-based analysis by age, sex, and comorbidity. Int J Biometeorol. 2021 Dec;65(12):2087–98.

17. Lu P, Xia G, Zhao Q, Xu R, Li S, Guo Y. Temporal trends of the association between ambient temperature and hospitalisations for cardiovascular diseases in Queensland, Australia from 1995 to 2016: A time-stratified case-crossover study. PLoS Med. 2020 Jul 21;17(7):e1003176.

18. Zhan ZY, Zhong X, Yang J, Ding Z, Xie XX, Zheng ZQ, et al. Effect of apparent temperature on hospitalization from a spectrum of cardiovascular diseases in rural residents in Fujian, China. Environ Pollut. 2022 Jun 15;303:119101.

19. Xu Z, Tong S, Pan H, Cheng J. Associations of extreme temperatures with hospitalizations and post-discharge deaths for stroke: What is the role of pre-existing hyperlipidemia? Environ Res. 2021 Feb;193:110391.

20. Wichmann J, Rosengren A, Sjöberg K, Barregard L, Sallsten G. Association between ambient temperature and acute myocardial infarction hospitalisations in Gothenburg, Sweden: 1985-2010. PloS One. 2013;8(4):e62059.

21. Yu X, Xia L, Xiao J, Zheng J, Xu N, Feng X, et al. Association of Daily Mean Temperature and Temperature Variability With Onset Risks of Acute Aortic Dissection. J Am Heart Assoc. 2021 Jul 6;10(13):e020190.

22. Lam HCY, Chan JCN, Luk AOY, Chan EYY, Goggins WB. Short-term association between ambient temperature and acute myocardial infarction hospitalizations for diabetes mellitus patients: A time series study. PLoS Med. 2018 Jul;15(7):e1002612.

23. Wichmann J, Andersen ZJ, Ketzel M, Ellermann T, Loft S. Apparent Temperature and Cause-Specific Mortality in Copenhagen, Denmark: A Case-Crossover Analysis. Int J Environ Res Public Health. 2011 Sep;8(9):3712–27.

24. Wichmann J, Ketzel M, Ellermann T, Loft S. Apparent temperature and acute myocardial infarction hospital admissions in Copenhagen, Denmark: a case-crossover study. Environ Health. 2012 Mar 30;11:19.

25. Ge Y, Liu C, Niu Y, Chen C, Wang W, Lin Z, et al. Associations between ambient temperature and daily hospital admissions for rheumatic heart disease in Shanghai, China. Int J Biometeorol. 2018 Dec 1;62(12):2189–95.

26. Abrutzky R, Dawidowski L, Matus P, Lankao PR. Health Effects of Climate and Air Pollution in Buenos Aires: A First Time Series Analysis. J Environ Prot. 2012 Mar 20;3(3):262–71.

27. Lu P, Zhao Q, Xia G, Xu R, Hanna L, Jiang J, et al. Temporal trends of the association between ambient temperature and cardiovascular mortality: a 17-year case-crossover study. Environ Res Lett. 2021 Mar;16(4):045004.

28. Saucy A, Ragettli MS, Vienneau D, de Hoogh K, Tangermann L, Schäffer B, et al. The role of extreme temperature in cause-specific acute cardiovascular mortality in Switzerland: A case-crossover study. Sci Total Environ. 2021 Oct 10;790:147958.

29. Xu R, Huang S, Shi C, Wang R, Liu T, Li Y, et al. Extreme Temperature Events, Fine Particulate Matter, and Myocardial Infarction Mortality. Circulation. 2023 Jul 25;148(4):312–23.

30. Zhou L, Chen K, Chen X, Jing Y, Ma Z, Bi J, et al. Heat and mortality for ischemic and hemorrhagic stroke in 12 cities of Jiangsu Province, China. Sci Total Environ. 2017 Dec 1;601–602:271–7.

31. Wichmann J, Andersen Z, Ketzel M, Ellermann T, Loft S. Apparent Temperature and Cause-Specific Emergency Hospital Admissions in Greater Copenhagen, Denmark. PLoS ONE. 2011 Jul 29;6(7):e22904.

32. Huang J, Wang J, Yu W. The Lag Effects and Vulnerabilities of Temperature Effects on Cardiovascular Disease Mortality in a Subtropical Climate Zone in China. Int J Environ Res Public Health. 2014 Apr;11(4):3982–94.

33. Zhai G, Tian Y, Zhang K, Qi J, Chai G. The effect of apparent temperature on hospital admissions for cardiovascular diseases in rural areas of Pingliang, China. Ann Agric Environ Med AAEM. 2022 Jun 24;29(2):281–6.

34. Ikefuti PV, Barrozo LV, Braga ALF. Mean air temperature as a risk factor for stroke mortality in São Paulo, Brazil. Int J Biometeorol. 2018 Aug 1;62(8):1535–42.

35. Li J, Xu X, Yang J, Liu Z, Xu L, Gao J, et al. Ambient high temperature and mortality in Jinan, China: A study of heat thresholds and vulnerable populations. Environ Res. 2017 Jul 1;156:657–64.

36. Li M, Shaw BA, Zhang W, Vásquez E, Lin S. Impact of Extremely Hot Days on Emergency Department Visits for Cardiovascular Disease among Older Adults in New York State. Int J Environ Res Public Health. 2019 Jan;16(12):2119.

37. Ma Y, Jiao H, Zhang Y, Feng F, Cheng B, Ma B, et al. Short-term effect of extreme air temperature on hospital emergency room visits for cardiovascular diseases from 2009 to 2012 in Beijing, China. Environ Sci Pollut Res. 2020 Oct;27(30):38029–37.

38. Shen Y, Zhang X, Chen C, Lin Q, Li X, Qu W, et al. The relationship between ambient temperature and acute respiratory and cardiovascular diseases in Shenyang, China. Environ Sci Pollut Res Int. 2021;28(16):20058–71.

39. Lin S, Luo M, Walker RJ, Liu X, Hwang SA, Chinery R. Extreme high temperatures and hospital admissions for respiratory and cardiovascular diseases. Epidemiol Camb Mass. 2009 Sep;20(5):738–46.

40. Zhai L, Ma X, Wang J, Luan G, Zhang H. Effects of ambient temperature on cardiovascular disease: a time-series analysis of 229288 deaths during 2009-2017 in Qingdao, China. Int J Environ Health Res. 2022 Jan;32(1):181–90.

41. Rowland ST, Boehme AK, Rush J, Just AC, Kioumourtzoglou MA. Can ultra short-term changes in ambient temperature trigger myocardial infarction? Environ Int. 2020 Oct;143:105910.

42. Cho SK, Sohn J, Cho J, Noh J, Ha KH, Choi YJ, et al. Effect of Socioeconomic Status and Underlying Disease on the Association between Ambient Temperature and Ischemic Stroke. Yonsei Med J. 2018 Jul 1;59(5):686–92.

43. Li N, Ma J, Liu F, Zhang Y, Ma P, Jin Y, et al. Associations of apparent temperature with acute cardiac events and subtypes of acute coronary syndromes in Beijing, China. Sci Rep. 2021 Jul 27;11:15229.

44. Liu X, Kong D, Fu J, Zhang Y, Liu Y, Zhao Y, et al. Association between extreme temperature and acute myocardial infarction hospital admissions in Beijing, China: 2013–2016. PLoS ONE. 2018 Oct 17;13(10):e0204706.

45. Han MH, Yi HJ, Ko Y, Kim YS, Lee YJ. Association between hemorrhagic stroke occurrence and meteorological factors and pollutants. BMC Neurol. 2016 May 4;16:59.

46. Gebhard C, Gebhard CE, Stähli BE, Maafi F, Bertrand MJ, Wildi K, et al. Weather and risk of ST-elevation myocardial infarction revisited: Impact on young women. PLoS ONE. 2018 Apr 9;13(4):e0195602.

47. Xing Q, Sun Z, Tao Y, Zhang X, Miao S, Zheng C, et al. Impacts of urbanization on the temperature-cardiovascular mortality relationship in Beijing, China. Environ Res. 2020 Dec;191:110234.

48. Tian Z, Li S, Zhang J, Jaakkola JJ, Guo Y. Ambient temperature and coronary heart disease mortality in Beijing, China: a time series study. Environ Health. 2012 Aug 21;11(1):56.

49. Grjibovski AM, Nurgaliyeva N, Kosbayeva A, Sharbakov A, Seysembekov T, Menne B. Effect of High Temperatures on Daily Counts of Mortality from Diseases of Circulatory System in Astana, Kazakhstan. Medicina (Mex). 2012 Dec;48(12):94.

50. Wang Y, Liu Y, Ye D, Li N, Bi P, Tong S, et al. High temperatures and emergency department visits in 18 sites with different climatic characteristics in China: Risk assessment and attributable fraction identification. Environ Int. 2020 Mar 1;136:105486.

51. Cleland SE, Steinhardt W, Neas LM, Jason West J, Rappold AG. Urban heat island impacts on heat-related cardiovascular morbidity: A time series analysis of older adults in US metropolitan areas. Environ Int. 2023 Aug 1;178:108005.

52. Jiang Y, Hu J, Peng L, Li H, Ji JS, Fang W, et al. Non-optimum temperature increases risk and burden of acute myocardial infarction onset: A nationwide case-crossover study at hourly level in 324 Chinese cities. EClinicalMedicine. 2022 Aug;50:101501.

53. Lee S, Lee E, Park MS, Kwon BY, Kim H, Jung DH, et al. Short-Term Effect of Temperature on Daily Emergency Visits for Acute Myocardial Infarction with Threshold Temperatures. PLoS ONE. 2014 Apr 25;9(4):e94070.

54. Son JY, Bell ML, Lee JT. The impact of heat, cold, and heat waves on hospital admissions in eight cities in Korea. Int J Biometeorol. 2014 Nov 1;58(9):1893–903.

55. Kwon BY, Lee E, Lee S, Heo S, Jo K, Kim J, et al. Vulnerabilities to Temperature Effects on Acute Myocardial Infarction Hospital Admissions in South Korea. Int J Environ Res Public Health. 2015 Nov;12(11):14571–88.

56. Chen R, Li T, Cai J, Yan M, Zhao Z, Kan H. Extreme temperatures and out-of-hospital coronary deaths in six large Chinese cities. J Epidemiol Community Health. 2014 Dec;68(12):1119–24.

57. Lim YH, Kim H, Hong YC. Variation in mortality of ischemic and hemorrhagic strokes in relation to high temperature. Int J Biometeorol. 2013 Jan 1;57(1):145–53.

58. Wang XY, Guo Y, FitzGerald G, Aitken P, Tippett V, Chen D, et al. The Impacts of Heatwaves on Mortality Differ with Different Study Periods: A Multi-City Time Series Investigation. PLoS ONE. 2015 Jul 28;10(7):e0134233.

59. Ban J, Xu D, He MZ, Sun Q, Chen C, Wang W, et al. The effect of high temperature on cause-specific mortality: A multi-county analysis in China. Environ Int. 2017 Sep 1;106:19–26.

60. Onozuka D, Hagihara A. Out-of-hospital cardiac arrest risk attributable to temperature in Japan. Sci Rep. 2017 Jan 3;7:39538.

61. Basu R, Ostro BD. A Multicounty Analysis Identifying the Populations Vulnerable to Mortality Associated with High Ambient Temperature in California. Am J Epidemiol. 2008 Sep 15;168(6):632–7.

62. Phung D, Guo Y, Thai P, Rutherford S, Wang X, Nguyen M, et al. The effects of high temperature on cardiovascular admissions in the most populous tropical city in Vietnam. Environ Pollut Barking Essex 1987. 2016 Jan;208(Pt A):33–9.

63. Phung D, Chu C, Rutherford S, Nguyen HLT, Do CM, Huang C. Heatwave and risk of hospitalization: A multi-province study in Vietnam. Environ Pollut. 2017 Jan 1;220:597–607.

64. Campbell SL, Remenyi TA, Williamson GJ, White CJ, Johnston FH. The Value of Local Heatwave Impact Assessment: A Case-Crossover Analysis of Hospital Emergency Department Presentations in Tasmania, Australia. Int J Environ Res Public Health. 2019 Jan;16(19):3715.

65. Ha S, Talbott EO, Kan H, Prins CA, Xu X. The effects of heat stress and its effect modifiers on stroke hospitalizations in Allegheny County, Pennsylvania. Int Arch Occup Environ Health. 2014 Jul;87(5):557–65.

66. Fisher JA, Jiang C, Soneja SI, Mitchell C, Puett RC, Sapkota A. Summertime extreme heat events and increased risk of acute myocardial infarction hospitalizations. J Expo Sci Environ Epidemiol. 2017 May;27(3):276–80.

67. Huang W, Kan H, Kovats S. The impact of the 2003 heat wave on mortality in Shanghai, China. Sci Total Environ. 2010 May 1;408(11):2418–20.

68. Zeng W, Lao X, Rutherford S, Xu Y, Xu X, Lin H, et al. The effect of heat waves on mortality and effect modifiers in four communities of Guangdong Province, China. Sci Total Environ. 2014 Jun 1;482–483:214–21.

69. Zacharias S, Koppe C, Mücke HG. Influence of Heat Waves on Ischemic Heart Diseases in Germany. Climate. 2014 Sep;2(3):133–52.

70. Moraes SL de, Almendra R, Barrozo LV. Impact of heat waves and cold spells on cause-specific mortality in the city of São Paulo, Brazil. Int J Hyg Environ Health. 2022 Jan 1;239:113861.

71. Urban A, Davídkovová H, Kyselý J. Heat- and cold-stress effects on cardiovascular mortality and morbidity among urban and rural populations in the Czech Republic. Int J Biometeorol. 2014 Aug 1;58(6):1057–68.

72. Dong W, Zeng Q, Ma Y, Li G, Pan X. Impact of Heat Wave Definitions on the Added Effect of Heat Waves on Cardiovascular Mortality in Beijing, China. Int J Environ Res Public Health. 2016 Sep;13(9):933.

73. Tian Z, Li S, Zhang J, Guo Y. The Characteristic of Heat Wave Effects on Coronary Heart Disease Mortality in Beijing, China: A Time Series Study. PLOS ONE. 2013 Sep 30;8(9):e77321.

74. Perčič S, Kukec A, Cegnar T, Hojs A. Number of Heat Wave Deaths by Diagnosis, Sex, Age Groups, and Area, in Slovenia, 2015 vs. 2003. Int J Environ Res Public Health. 2018 Jan 22;15(1):173.

75. Hanzlíková H, Plavcová E, Kynčl J, Kříž B, Kyselý J. Contrasting patterns of hot spell effects on morbidity and mortality for cardiovascular diseases in the Czech Republic, 1994-2009. Int J Biometeorol. 2015 Nov;59(11):1673–84.

76. Ponjoan A, Blanch J, Alves-Cabratosa L, Martí-Lluch R, Comas-Cufí M, Parramon D, et al. Effects of extreme temperatures on cardiovascular emergency hospitalizations in a Mediterranean region: a self-controlled case series study. Environ Health. 2017 Apr 4;16(1):32.

77. Fonseca-Rodríguez O, Sheridan SC, Lundevaller EH, Schumann B. Effect of extreme hot and cold weather on cause-specific hospitalizations in Sweden: A time series analysis. Environ Res. 2021 Feb;193:110535.

78. Oudin Åström D, Schifano P, Asta F, Lallo A, Michelozzi P, Rocklöv J, et al. The effect of heat waves on mortality in susceptible groups: a cohort study of a mediterranean and a northern European City. Environ Health. 2015 Mar 29;14(1):30.

79. D’Ippoliti D, Michelozzi P, Marino C, de’Donato F, Menne B, Katsouyanni K, et al. The impact of heat waves on mortality in 9 European cities: results from the EuroHEAT project. Environ Health Glob Access Sci Source. 2010 Jul 16;9:37.
